# Role of electroanatomical mapping–guided superior vena cava isolation in paroxysmal atrial fibrillation patients without provoked superior vena cava triggers: a randomized controlled study

**DOI:** 10.1093/europace/euae039

**Published:** 2024-02-02

**Authors:** Yan Dong, Dongsheng Zhao, Xinguang Chen, Linshen Shi, Qiushi Chen, Haiyan Zhang, Yue Yu, Inam Ullah, Pipin Kojodjojo, Fengxiang Zhang

**Affiliations:** Section of Pacing and Electrophysiology, Division of Cardiology, The First Affiliated Hospital with Nanjing Medical University, Guangzhou Road 300, Nanjing 210029, China; Section of Pacing and Electrophysiology, Division of Cardiology, The First Affiliated Hospital with Nanjing Medical University, Guangzhou Road 300, Nanjing 210029, China; Section of Pacing and Electrophysiology, Division of Cardiology, The First Affiliated Hospital with Nanjing Medical University, Guangzhou Road 300, Nanjing 210029, China; Section of Pacing and Electrophysiology, Division of Cardiology, The First Affiliated Hospital of Gannan Medical University, Ganzhou, China; Department of Cardiology, The Second Affiliated Hospital of Nantong University, Nantong, China; Section of Pacing and Electrophysiology, Division of Cardiology, The First Affiliated Hospital with Nanjing Medical University, Guangzhou Road 300, Nanjing 210029, China; Department of Cardiology, The Second Affiliated Hospital of Nanjing Medical University, Nanjing, China; Section of Pacing and Electrophysiology, Division of Cardiology, The First Affiliated Hospital with Nanjing Medical University, Guangzhou Road 300, Nanjing 210029, China; Section of Pacing and Electrophysiology, Division of Cardiology, The First Affiliated Hospital with Nanjing Medical University, Guangzhou Road 300, Nanjing 210029, China; Asian Heart and Vascular Centre, National University of Singapore, Singapore, Singapore; Section of Pacing and Electrophysiology, Division of Cardiology, The First Affiliated Hospital with Nanjing Medical University, Guangzhou Road 300, Nanjing 210029, China

**Keywords:** Atrial fibrillation, Catheter ablation, Superior vena cava

## Abstract

**Aims:**

Data about whether empirical superior vena cava (SVC) isolation (SVCI) improves the success rate of paroxysmal atrial fibrillation (PAF) are conflicting. This study sought to first investigate the characteristics of SVC-triggered atrial fibrillation and secondly investigate the impact of electroanatomical mapping–guided SVCI, in addition to circumferential pulmonary vein isolation (CPVI), on the outcome of PAF ablation in the absence of provoked SVC triggers.

**Methods and results:**

A total of 130 patients undergoing PAF ablation underwent electrophysiological studies before ablation. In patients for whom SVC triggers were identified, SVCI was performed in addition to CPVI. Patients without provoked SVC triggers were randomized in a 1:1 ratio to CPVI plus SVCI or CPVI only. The primary endpoint was freedom from any documented atrial tachyarrhythmias lasting over 30 s after a 3-month blanking period without anti-arrhythmic drugs at 12 months after ablation. Superior vena cava triggers were identified in 30 (23.1%) patients with PAF. At 12 months, 93.3% of those with provoked SVC triggers who underwent CPVI plus SVCI were free from atrial tachyarrhythmias. In patients without provoked SVC triggers, SVCI, in addition to CPVI, did not increase freedom from atrial tachyarrhythmias (87.9 vs. 79.6%, log-rank *P* = 0.28).

**Conclusion:**

Electroanatomical mapping–guided SVCI, in addition to CPVI, did not increase the success rate of PAF ablation in patients who had no identifiable SVC triggers.

**Registration:**

ChineseClinicalTrials.gov: ChiCTR2000034532

## Graphical Abstract

**Graphical Abstract:**
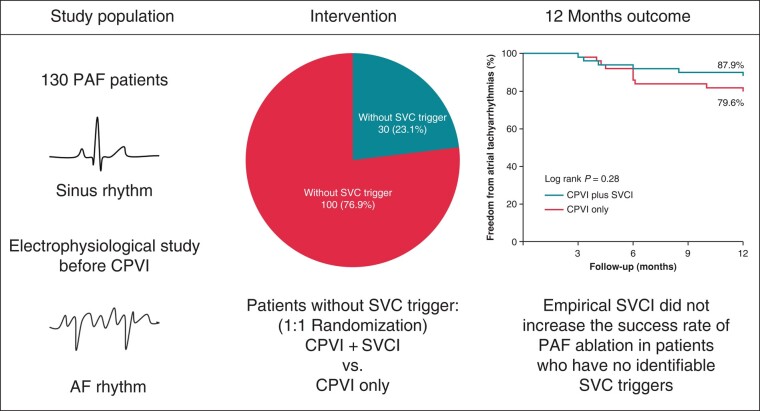
Role of empirical SVCI in PAF patients without provoked SVC triggers. CPVI, circumferential pulmonary vein isolation; PAF, paroxysmal atrial fibrillation; SVC, superior vena cava; SVCI, SVC isolation.

**What’s new?**

- Empirical superior vena cava (SVC) isolation (SVCI), in addition to circumferential pulmonary vein isolation (CPVI), did not increase the success rate for patients with paroxysmal atrial fibrillation (PAF) in the absence of identifiable SVC triggers.
- This is the first randomized controlled study to compare the 12-month success rate of CPVI plus SVCI vs. CPVI only for patients with PAF in the absence of identifiable SVC triggers.
- Different from previous studies, induction for triggers was performed before CPVI in the present study. Superior vena cava–triggered atrial fibrillation was identified in 23.1% of patients.

## Introduction

Circumferential pulmonary vein (PV) isolation (CPVI) has proven to be the cornerstone of radiofrequency catheter ablation of atrial fibrillation (AF).^1,2^ However, non-PV triggers also play an important role in initiating AF in 10–20% of patients.^3–5^ The common non-PV triggers include the superior vena cava (SVC), coronary sinus (CS) ostium, left atrial posterior wall, left atrial appendage, ligament of Marshall, inter-atrial septum, and crista terminalis.^6–8^ The SVC is a major origin of non-PV triggers accounting for 25–40%,^9–11^ which may be associated with the heterogeneity of the SVC sleeve and the arrhythmogenicity of cardiomyocytes isolated from the SVC.^9,12^

Whether SVC isolation (SVCI) is necessary remains controversial.^13–15^ The objective of this randomized controlled study was to investigate the impact of electroanatomical mapping–guided SVCI, in addition to CPVI, on the outcome of paroxysmal AF (PAF) ablation when SVC triggers cannot be provoked or identified. The study was carried out in accordance with the principles of the Declaration of Helsinki and was approved by the Ethics Committee. All subjects were given written informed consent before participating in the study. This study was registered with the Chinese Clinical Trial Registry (ChiCTR2000034532).

## Methods

### Study population

This prospective, multi-centre, randomized controlled study was conducted in four electrophysiology centres (three in China and one in Singapore). The study population included patients with symptomatic drug-refractory PAF referred for catheter ablation between December 2019 and June 2022. Exclusion criteria included age <18 or >80 years, prior AF ablation, and severe structural heart diseases (including hypertrophic cardiomyopathy, dilated cardiomyopathy, and valvular heart disease).

**Figure 1:**
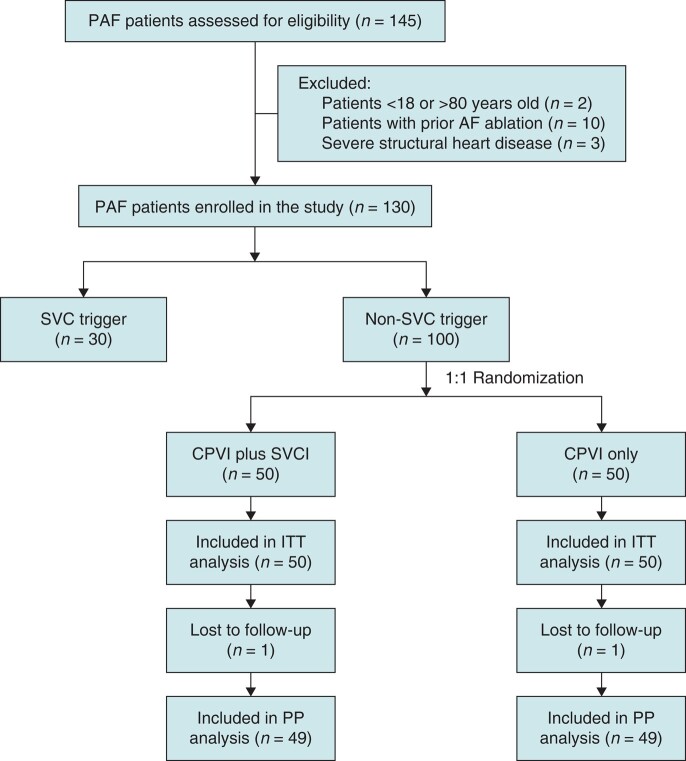
A flow chart of the study. AF, atrial fibrillation; CPVI, circumferential pulmonary vein isolation; ITT, intention-to-treat; PAF, paroxysmal atrial fibrillation; PP, per-protocol; SVC, superior vena cava; SVCI, SVC isolation.

Patients with SVC triggers received CPVI and SVCI. The remaining patients without SVC triggers were randomized 1:1 to CPVI plus SVCI or CPVI-only groups. The flow chart of the study is described in *Figure 1*.

### Peri-operative management and mapping

**Figure 2:**
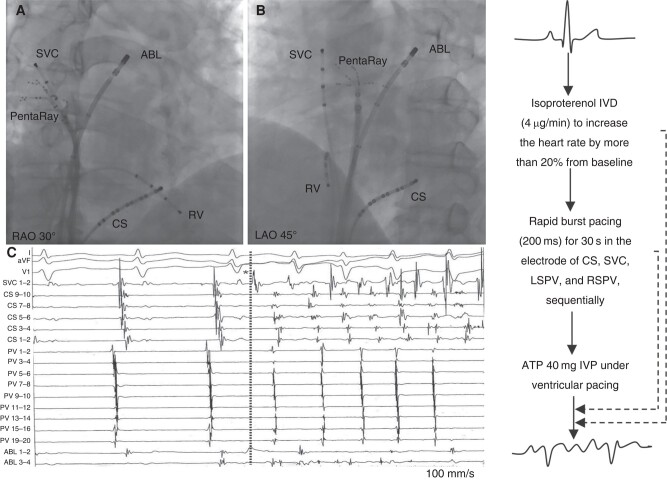
The fluoroscopy images of catheter setting during the electrophysiological studies. (*A*: RAO, right anterior oblique 30° and *B*: LAO, left anterior oblique 45°) The catheter PentaRay and the ABL are placed in the RSPV and LSPV, respectively. A 6 Fr decapolar catheter and two 6 Fr quadripolar catheters were positioned in the CS, RV, and SVC, respectively. (*C*) An example of AF originating from SVC. The symbol (*) represents the initiation of AF. ABL, ablation catheter; AF, atrial fibrillation; ATP, adenosine triphosphate; CS, coronary sinus; IVD, intravenous drip; IVP, intravenous push; LSPV, left superior pulmonary vein; RSPV, right superior pulmonary vein; RV, right ventricular; SVC, superior vena cava.

Before the procedure, all anti-arrhythmic agents, except amiodarone, were withdrawn for at least five half-lives, and patients received uninterrupted oral anticoagulants for at least 3 weeks. Left atrial thrombi were ruled out by transoesophageal echocardiography or cardiac computed tomography. The procedure was performed under conscious sedation with intravenous fentanyl. A 6 Fr decapolar catheter and two 6 Fr quadripolar catheters were positioned in the CS, right ventricular apex, and SVC, respectively, for pacing and recording via the femoral vein (*Figure 2*). After a double transseptal access into the left atrium (LA), a multi-polar catheter (PentaRay; Biosense Webster, Diamond Bar, CA, USA) was inserted through the long sheath to map the PV ostia, and a 3.5 mm irrigated tip ablation catheter (Thermocool Smarttouch; Biosense Webster) was prepared for ablation. All ablation procedures were guided by a 3D electroanatomical mapping system (CARTO 3, Biosense Webster). During the procedure, intravenous heparin was administered to maintain an activated clotting time between 300 and 350 s.

### Electrophysiological studies for the induction of atrial fibrillation triggers

Before ablation, electrophysiological studies (EPSs) were performed to identify the site of the origin initiating AF. The multi-polar catheter PentaRay and the ablation catheter Thermocool Smarttouch were placed in the right superior PV (RSPV) and left superior PV (LSPV), respectively (*Figure 2*). Isoproterenol (ISP) was intravenously injected (4 μg/min) to increase the heart rate by more than 20% from baseline. If no spontaneous AF occurred, rapid burst pacing (200 ms) for 30 s was performed in the electrode of the CS, SVC, LSPV, and RSPV, sequentially. If AF was induced, the next step was to observe the initial beat at the onset of the induced AF. If AF was not induced after these steps, boluses of adenosine triphosphate (ATP, 40 mg) were administered during ventricular pacing (500 ms) to uncover the ectopic beat initiating AF. Additional radiofrequency applications were applied to eliminate non-PV foci after CPVI.

### Circumferential pulmonary vein isolation

For CPVI, radiofrequency energy was set up at a power of 35–40 W, contact force of 10–20 g, and flow rate of 25–30 mL/min. The target ablation index was 500 for the anterior walls, while the target ablation index was 350–400 for the posterior walls. The endpoint of CPVI was defined as the achievement of the bidirectional conduction block between the PVs and the LA, after 30 min of waiting time, and the presence of ISP and ATP. If AF reinduced, it continued to look for the trigger foci and ablated it.

### Superior vena cava isolation

The right atrium was anatomically constructed using the multi-polar catheter PentaRay. The location of the sinus node (SN) was identified by using an activation map during sinus rhythm to prevent SN injury. Local maximum output pacing (20 mA) was performed at the free wall of the SVC to tag the location of the phrenic nerve.^16^ The SVC–right atrial (RA) junction was defined as the horizontal line at the ostium of the SVC, where the diameter of the SVC increased abruptly compared with the RA geometry.^17^ The following parameters were measured: the length from the SVC–RA junction to the distal SVC myocardial sleeve, the length from the SN to the distal SVC myocardial sleeve, and the diameter of the SVC in the SVC–RA junction. The following parameters were also measured: the length from the SVC–RA junction to the most distal SVC myocardial sleeve, the length from the SN to the distal SVC myocardial sleeve, and the diameter of the SVC in the SVC–RA junction. Based on the location of the SN, patients were divided into the In-SVC group and the In-RA group. If the earliest activation site was located at the RA–SVC junction, it was classified as the RA part.^18^

Segmental ablation was performed at least 10 mm above the SN. Radiofrequency energy was delivered at 40 W with a flow rate of 30 mL/min for the septal wall and at 35 W with a flow rate of 25 mL/min for the free wall. The endpoint of SVCI was the entrance block. In addition, if atrial flutter was previously documented or induced, an additional cavo-tricuspid isthmus line ablation was performed.

### Follow-up

Patients continued anticoagulation therapy for at least 3 months after ablation. The anti-arrhythmic agent was used only for 3 months after ablation. Patients with recurrent atrial tachyarrhythmias continued to take anti-arrhythmic agents after 3 months. Follow-up visits were performed at 1, 3, 6, and 12 months and every 6 months thereafter. The 24 h Holter was performed at each follow-up visit, while 7-day Holter was arranged at 12 months.

### Endpoints

The primary endpoint was freedom from any documented atrial tachyarrhythmias (AF/atrial flutter/atrial tachycardia) lasting over 30 s after a 3-month blanking period without anti-arrhythmic drugs at 12 months after the procedure. The primary endpoint analysis was based on an intention-to-treat principle. The secondary endpoints of the study included the following: incidence of peri-procedural complications, procedure time, fluoroscopy time, and total radiofrequency delivery time.

### Statistical analysis

Continuous variables with a normal distribution were presented as mean ± standard deviation and were compared using the two-sample *t-*test. Non-normally distributed continuous variables were presented as median with inter-quartile range and were compared using the Mann–Whitney *U* test. Categorical variables were presented as frequency (percentage) and were analysed using the *χ*^2^ test or Fisher’s exact test. Kaplan–Meier survival curves showed survival rate freedom from atrial tachyarrhythmias using the log-rank test. A two-sided *P*-value <0.05 was considered statistically significant. In a randomized controlled trial of cryoballoon vs. radiofrequency ablation (CPVI only) for PAF, the estimated 12-month success rate for CPVI using radiofrequency was 64.1%.^19^ An improvement in the success rate to 88% was considered clinically significant. A sample size of 94 patients, randomized in a 1:1 ratio, would provide 80% power to detect a 24% increase in the primary endpoint, with an alpha of 0.05. Allowing for a 5% dropout rate, at least 100 patients should be randomized.

## Results

### Trigger foci of atrial fibrillation

**Figure 3:**
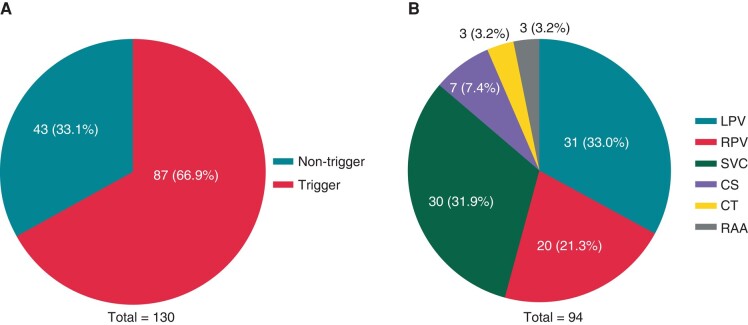
The incidence (*A*) and distribution (*B*) of trigger foci in 130 patients undergoing PAF ablation. (*A*) AF was induced in 87 (66.9%) patients. (*B*) Among 94 trigger foci in 87 patients, the trigger foci were the LPV (33.0%), SVC (31.9%), RPV (21.3%), CS ostium (7.4%), CT (3.2%), and RAA (3.2%), respectively. AF, atrial fibrillation; CS, coronary sinus; CT, crista terminalis; LPV, left pulmonary vein; PAF, paroxysmal AF; RAA, right atrial appendage; RPV, right pulmonary vein; SVC, superior vena cava.

Among 130 patients enrolled in this study, the PV trigger foci were observed in 39.2% (51/130) of patients. Non-PV trigger foci were observed in 33.1% (43/130) of patients, including 23.1% (30/130) of SVC-triggered AF patients. Atrial fibrillation was induced in 87 (66.9%) patients (*Figure 3A*). There were 94 trigger foci in these 87 patients (*Figure 3B*). The trigger foci were the LPV (31, 33.0%), SVC (30, 31.9%), RPV (20, 21.3%), CS ostium (7, 7.4%), crista terminalis (3, 3.2%), and RA appendage (3, 3.2%), respectively.

### Baseline characteristics

All 30 patients with SVC triggers underwent CPVI and SVCI. The remaining 100 patients were randomized to CPVI plus SVCI (*n* = 50) or CPVI only (*n* = 50). Baseline characteristics were not significantly different between the CPVI plus SVCI and the CPVI-only groups, except for body mass index (BMI) and the use of a calcium channel blocker (*Table 1*). The BMI in the CPVI plus SVCI group was higher than that in the CPVI-only group (25.5 ± 2.7 vs. 24.3 ± 2.9, *P* = 0.03). The calcium channel blocker usage in the CPVI plus SVCI group was higher than that in the CPVI-only group (28.0 vs. 8.0%, *P* = 0.01).

**Table 1:** Baseline characteristics of patients in the CPVI plus SVCI group and the CPVI-only group

	CPVI plus SVCI (*n* = 50)	CPVI only (*n* = 50)	*P*-value
Age (years)	59.1 ± 8.9	57.1 ± 9.9	0.30
Male (%)	35 (70.0%)	37 (74.0%)	0.66
BMI (kg/m^2^)	25.5 ± 2.7	24.3 ± 2.9	0.03
Duration (months)	24 (11–60)	24 (6–72)	0.97
CHA_2_DS_2_-VASc score	1 (0–2)	1 (0–2)	0.06
HAS-BLED score	1 (1–2)	1 (0–1)	0.10
Smoke (%)	14 (28.0%)	13 (26.0%)	0.82
Alcohol (%)	11 (22.0%)	9 (18.0%)	0.62
Comorbidities (%)			
Hypertension	25 (50.0%)	19 (38.0%)	0.23
Diabetes mellitus	6 (12.0%)	4 (8.0%)	0.51
Coronary heart disease	5 (10.0%)	2 (4.0%)	0.44
Ischaemic stroke	2 (4.0%)	0 (0.0%)	0.50
Medication use (%)			
Beta-blocker	18 (36.0%)	20 (40.0%)	0.68
CCB	14 (28.0%)	4 (8.0%)	0.01
ACEI/ARB	9 (18.0%)	9 (18.0%)	1.00
Statin	14 (28.0%)	8 (16.0%)	0.15
LAD (mm)	38.3 ± 4.3	37.4 ± 2.9	0.21
RAD (mm)	34.1 ± 3.5	34.1 ± 3.4	0.10
LVDD (mm)	47.8 ± 3.9	48.1 ± 3.5	0.73
LVDS (mm)	31.9 ± 2.2	31.5 ± 2.5	0.44
LVEF (%)	62.8 ± 1.9	63.5 ± 3.1	0.23

ACEI, angiotensin converting enzyme inhibitor; ARB, angiotensin receptor blocker; BMI, body mass index; CCB, calcium channel blocker; CPVI, circumferential pulmonary vein isolation; LAD, left atrium diameter; LVDD, left ventricular end-diastolic diameter; LVDS, left ventricular end-systolic diameter; LVEF, left ventricular ejection fraction; RAD, right atrium diameter; SVC, superior vena cava; SVCI, SVC isolation.

### Electrophysiological parameters

Electrophysiological characteristics of the patients without provoked SVC triggers randomized to the CPVI plus SVCI group vs. the CPVI-only group were comparable (*Table 2*). The electrophysiological characteristics were comparable between patients with and without SVC triggers, except for the length of the SN to the distal SVC muscle sleeve (*Table 3*). The length of the SN to the distal SVC muscle sleeve in patients with SVC triggers was significantly longer than that in patients without SVC triggers (40.2 ± 14.5 vs. 31.7 ± 10.4, *P* = 0.02).

**Table 2:** Electrophysiological characteristics of patients in the CPVI plus SVCI group and the CPVI-only group

	CPVI plus SVCI (*n* = 50)	CPVI only (*n* = 50)	*P*-value
Left atrial volume (mm^3^)	103.9 ± 28.9	94.7 ± 17.7	0.09
Right atrial volume (mm^3^)	80.4 ± 27.1	79.5 ± 27.7	0.90
Diameter of the SVC port (mm)	20.8 ± 4.3	21.5 ± 4.5	0.47
Muscle sleeve length of the SVC (mm)			
Septum	38.5 ± 11.1	39.2 ± 11.0	0.81
Free wall	38.3 ± 11.2	40.3 ± 11.0	0.49
Length of the sinus node to the distal SVC muscle sleeve (mm)	31.6 ± 10.3	31.8 ± 10.7	0.94
Location of the sinus node (%) (*n* = 85)			0.61
In-RA	26 (59.1%)	22 (53.7%)	
In-SVC	18 (40.9%)	19 (46.3%)	

CPVI, circumferential pulmonary vein isolation; RA, right atrium; SVC, superior vena cava; SVCI, SVC isolation.

**Table 3:** Electrophysiological characteristics in patients with or without SVC triggers

	SVC trigger (*n* = 30)	Non-SVC trigger (*n* = 100)	*P*-value
Left atrial volume (mm^3^)	94.2 ± 31.1	99.3 ± 24.2	0.39
Right atrial volume (mm^3^)	79.8 ± 18.7	79.9 ± 27.2	0.99
Diameter of the SVC port (mm)	20.7 ± 3.5	21.1 ± 4.4	0.65
Muscle sleeve length of the SVC (mm)			
Septum	37.6 ± 11.2	38.9 ± 10.9	0.70
Free wall	37.8 ± 13.1	39.3 ± 11.0	0.66
Length of the sinus node to the distal SVC muscle sleeve (mm)	40.2 ± 14.5	31.7 ± 10.4	0.02
Location of the sinus node (%) (*n* = 114)			0.66
In-RA	15 (51.7%)	48 (56.5%)	
In-SVC	14 (48.3%)	37 (43.5%)	

RA, right atrium; SVC, superior vena cava.

### Efficacy

**Figure 4:**
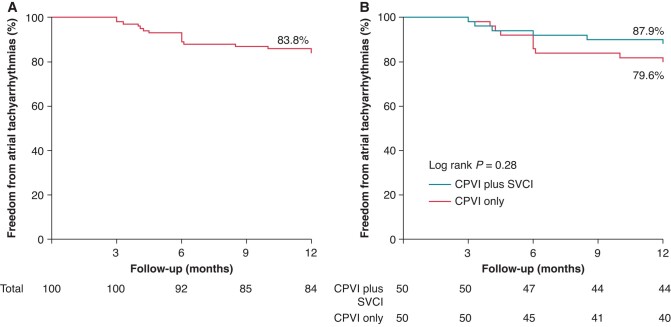
Kaplan–Meier survival rates freedom from atrial tachyarrhythmias without anti-arrhythmic drugs in randomized patients (*A*) and in patients in the CPVI plus SVCI group and the CPVI-only group (*B*). (*A*) The overall survival rate from atrial tachyarrhythmias was 83.8% among randomized patients. (*B*) The survival rates freedom from atrial tachyarrhythmias in the CPVI plus SVCI group and the CPVI-only group were 87.9 and 79.6%, respectively (log-rank *P* = 0.28). CPVI, circumferential pulmonary vein isolation; SVCI, superior vena cava isolation.

At 12 months after the procedure, 93.3% of those with provoked SVC triggers who underwent SVCI plus CPVI were free from atrial tachyarrhythmias. The overall survival rate from atrial tachyarrhythmias was 83.8% among the randomized patients (*Figure 4A*). A total of 16 patients (16%) had atrial tachyarrhythmia recurrences after a 3-month blanking period, 6 in the CPVI plus SVCI group and 10 in the CPVI-only group. The Kaplan–Meier analysis showed that the survival rates freedom from atrial tachyarrhythmias in the CPVI plus SVCI and CPVI-only groups were 87.9 and 79.6%, respectively (log-rank *P* = 0.28; *Figure 4B*). The ablation of non-PV triggers, except the SVC, was comparable between the two groups (8.0 vs. 12.0%, *P* = 0.51).

### Safety

No significant differences in procedure, fluoroscopy, and total ablation time were observed between the CPVI plus SVCI and the CPVI-only groups. No SN injury, phrenic nerve injury, and pericardial effusion occurred in the present study (*Table 4*).

**Table 4:** Procedural characteristics of patients in the CPVI plus SVCI group and the CPVI-only group

	CPVI plus SVCI (*n* = 50)	CPVI only (*n* = 50)	*P*-value
Procedure time (min)	200.2 ± 37.2	208.3 ± 48.1	0.35
Fluoroscopy time (min)	5.8 (2.3–10.0)	5.5 (3.0–11.0)	0.61
Total ablation time (min)	54.0 (47.3–60.0)	53.0 (42.0–59.0)	0.22
Ablation of non-PV triggers, except the SVC (%)	4 (8.0%)	6 (12.0%)	0.51
Peri-operative complications (%)			
Pericardial effusion	0	0	–
Thrombo-embolic events	0	0	–
Phrenic nerve injury	0	0	–
Sinus node injury	0	0	–
Death	0	0	–

CPVI, circumferential pulmonary vein isolation; PV, pulmonary vein; SVC, superior vena cava; SVCI, SVC isolation.

## Discussion

### Major findings

The main findings of this randomized controlled study were as follows: first, the SVC was the most common non-PV trigger foci in our subjects; secondly, the patients with SVC triggers had longer SVC muscle sleeves compared with those without SVC triggers; thirdly, electroanatomical mapping–guided SVCI avoided SN injury; and finally, SVCI in the absence of SVC triggers did not increase the success rate of PAF ablation.

### The induction of atrial fibrillation non-pulmonary vein triggers

In the present study, non-PV triggers were induced in 33.1% of patients, which was similar to previous studies.^3,20^ Aggressive stimulation for triggers was performed before CPVI and at the end of the ablation procedure in the present study. Although the induction protocol in the present study was similar to previous studies, the incidence of SVC-triggered AF (23.1%) in the present study was higher than that in previous studies.^21,22^ Takigawa *et al.*^21^ and Lin *et al.*^22^ reported the SVC-triggered AF rate of 6.6 and 10% in patients with PAF, respectively. In these previous studies, EPS to identify AF trigger foci was performed only after CPVI. Thus, non-PV triggers, including the SVC, may be masked by autonomic effects of CPVI.

### Electrophysiological characteristics of superior vena cava–triggered patients

The SVC, as the most common non-PV foci, contains cardiac muscles connected to the right atrium, and atrial excitation can propagate into the SVC.^23^ Superior vena cava cardiomyocytes were found to have pacemaker activity, and the enhanced automaticity and afterdepolarization play a role in the arrhythmogenic activity of the SVC.^24^ Previous studies have shown that SVC firing is more likely to occur in patients with long SVC sleeves (>30 mm) and large SVC potentials (>1 mV).^9,25^ A recent study conducted by Nyuta *et al.*^11^ demonstrated that longer L-SVC (length from the top of the SN to the top of the myocardial sleeve of the SVC) was an independent risk factor for SVC firing in patients with AF who underwent ablation, which was consistent with the result of our study.

### Outcomes

The role of SVCI in PAF patients undergoing index ablation is currently controversial. In the present study, the success rate of AF ablation was the highest in patients who had identifiable SVC triggers and received SVCI. This observation confirms that SVCI is really necessary for patients in the presence of SVC triggers. However, in patients with the absence of provoked SVC triggers, the success rate was similar between the SVCI plus CPVI group and the CPVI-only group, which is consistent with some previous studies,^26–28^ although these studies included patients with SVC-triggered AF. On the other hand, other studies have shown a benefit.^13,15,29^ Corrado *et al.*^15^ conducted a randomized controlled study of empirical SVCI and found that empirical CPVI plus SVCI was superior to CPVI in PAF patients (90 vs. 77%, *P* = 0.04) with a follow-up of 12 months. But, patients with PAF were only a subgroup of 320 patients with PAF, persistent AF, and permanent AF in the overall study population, and ablation was carried out with non-irrigated ablation catheters. Ejima *et al.*^29^ compared the outcomes of empiric SVCI vs. as-needed SVCI (SVCI if SVC triggered) in 186 patients with PAF and reported a lower recurrence rate in the empiric SVCI group compared with as-needed SVCI (23 vs. 44%, *P* = 0.035). But, this study was a non-randomized retrospective study with inherent operator bias. All previous studies on SVCI were conducted in non-specific patients with PAF, whereas our study focused on PAF patients without provoked SVC triggers. The present study suggests that electroanatomical mapping–guided SVCI is not routinely required in patients without provoked SVC triggers.

### Safety

Potential complications related to SVCI include phrenic nerve injury, SN injury, and SVC stenosis. In the present study, electroanatomical mapping–guided SVCI could maximumly avoid SN and phrenic nerve injury. The previously reported incidence of phrenic nerve injury is low (0–5%) and usually transient.^29–31^ This proves that our strategy of searching for phrenic nerve capture by local maximum output pacing (20 mA) before ablation is a safe method of avoiding phrenic nerve injury. Liu *et al*.^32^ reported that the right phrenic nerve can be visualized by intracardiac echocardiography (ICE) in most patients, and phrenic nerve injury can be prevented by the guidance of ICE. It was reported that, among 354 patients with SVCI during AF ablation, the rate of SN injury was 1.1%.^33^ No SN injury occurred in patients with electroanatomical mapping–guided SVCI in the present study.

## Limitations

The ablation of non-PV triggers, except SVCI, may affect the outcome of patients between the two groups. However, the ablation of non-PV triggers was comparable between the two groups due to a randomized controlled study, and the influence of different non-PV triggers could be ignored. The recurrence rate of atrial tachyarrhythmias may have been underestimated because asymptomatic atrial tachyarrhythmia episodes may be undetected using 24 h or 7-day Holter compared with implantable loop recorders.

## Conclusions

The superior vena cava was the most common source of non-PV triggers in patients with PAF. Electroanatomical mapping–guided SVCI, in addition to CPVI, did not increase the success rate of PAF ablation in patients who had no identifiable SVC triggers.

## Data Availability

The data underlying this article will be shared on reasonable request to the corresponding author.
